# Reporting accuracy of rare event classifiers

**DOI:** 10.1038/s41746-018-0062-0

**Published:** 2018-10-10

**Authors:** Edieal Pinker

**Affiliations:** Yale University, 165 Whitney Ave, New Haven, CT 06520 USA

**Keywords:** Prognosis, Outcomes research, Risk factors

The article by Rajkomar et al, “Scalable and accurate deep learning with electronic health records” published on 8 May 2018 in *npj Digital Medicine*,^1^ describes an effort to automate the process of taking all the data in an EHR (Electronic Health Record) system, including free-text notes, and transforming it into a format that can then be fed into a deep learning algorithm for various predictions. The authors claim that the deep learning algorithm was able to predict 24-h mortality with an area under the receiver operating characteristic curve (AUROC) of 95%.

An AUROC of 95% is generally viewed as good performance in the literature, but it is unclear what it means from a clinical perspective. The predictive algorithms in ref. ^1^ and others that attempt to predict patient mortality are performing classification. Given a population, they assign each member of the patient population based on its characteristics; if the score is above a threshold they classify the patient as going to die soon or at high risk of short-term mortality. The patient population has two subgroups, those that are actually going to die in the time window of interest (subgroup A) and those that won’t (subgroup B). Each subgroup has its own probability distribution for the score that a patient in that subgroup will receive from the predictive algorithm. If the distributions have a lot of overlap it will be difficult to discriminate between the two with this scoring algorithm. The interpretation of the AUROC is that it gives the probability that a randomly selected patient from subgroup A will have a higher score than a randomly selected patient from subgroup B. Note that this comparison is not influenced by the overall prevalence of subgroup A in the population. Yet, if A is a rare event, such as the 2.3% 24-h mortality reported in ref. ^1^, it is possible that the right tail of the distribution of scores for subgroup B is of significance relative to the size of the entire population of subgroup A.

Most non-statisticians do not understand the AUROC and thus misinterpret it. This paper received wide coverage in the mainstream media. For example, Tung^2^ described its performance as follows: “On inpatient mortality, for example, it scored 0.95 out of a perfect score of 1.0 compared with traditional methods, which scored 0.86.”

This does not mean that a patient classified as going to die has a 95% chance of dying, but that is how the public interprets the results. We do not know what that probability is, because the positive predictive value (PPV) has not been reported; it could in fact be much lower because mortality is so rare in the data set. PPV, however, takes into account the overall prevalence of the subgroup A in the entire population.

It is becoming better accepted that AUROC is a poor standard for evaluating discrimination power for a classifier when the data are very imbalanced. As well described in ref. ^3^ and ^4^, the precision recall curve (PRC), defined as a plot of sensitivity versus PPV, and the corresponding area under the precision recall curve (AUPRC) are much more informative of the relevant accuracy of prediction methods than the AUROC. A recent example of applying this approach for mortality prediction for advanced cancer patients appears in ref. ^5^. The AUPRC does not have a natural interpretation, but it is clear that higher is better and each point on the PRC provides the clinician important information about a classification threshold: the fraction of the subgroup A patients that are identified and the fraction of those classified as subgroup A that are actually in subgroup A.

To illustrate the point we can conduct the following simulation exercise. Model subgroup A’s scores are normally distributed with mean 30 and standard deviation 10, and subgroup B's scores are normally distributed with mean 20 and standard deviation 10. A parameter (*p*) represents the fraction of the population that is expected to be in subgroup A. For a given value of *p*, randomly generate a population of patients by assigning each to subgroup A with probability *p* and drawing the A and B scores from the respective distributions. The ROC, PRC and respective areas under each of these curves can be generated for this population. The impact of prevalence on the performance can be shown by varying *p* from 0.5 to 0.025.

The simulation model was coded and executed in Matlab. Each simulation has a population of 1000 randomly generated samples, and is replicated 100 times for each value of *p*. The resulting average of AUROC and AUPRC is reported in Table 1. These data show that AUROC is insensitive to prevalence, while AUPRC declines sharply with prevalence, and the differences between AUROC and AUPRC are small when the subgroup sizes are similar. Therefore we cannot only rely on AUROC to evaluate the performance of classification algorithms when trying to predict relatively rare events. We also note that even if the algorithm in ref. ^1^ delivers a PPV of, say, 40% using a particular threshold, that could be quite informative because it is able to classify some patients as having a 40% chance of short-term mortality in a population with a base rate of only 2.3%. Indeed the value of this depends on the corresponding sensitivity. The value also depends upon something that is not discussed in ref.^1^ and is as yet very poorly understood. How many of the patients classified as high risk would not have been identified by the clinicians? Unfortunately, neither AUROC nor AUPRC tells us anything about that.

**Table 1:** Comparison of average AUROC and average AUPRC for different prevalence levels

Prevalence *p*	0.5	0.425	0.325	0.225	0.125	0.025
Avg. AUROC	0.759	0.762	0.760	0.760	0.757	0.755
StdErr AUROC	0.001	0.002	0.002	0.002	0.002	0.005
Avg. AUPRC	0.753	0.701	0.609	0.502	0.349	0.118
StdErr AUPRC	0.002	0.002	0.003	0.003	0.004	0.005

Thus, I recommend that researchers report the AUPRC for their studies involving classification to give a more realistic and less hype-able evaluation of accuracy.

## Code availability

Matlab code is available from the author.

## Data Availability

Data were generated via Monte-Carlo simulation as described in the article.
